# Preparation and Characterization of *O*-Acylated Fucosylated Chondroitin Sulfate from Sea Cucumber

**DOI:** 10.3390/md10081647

**Published:** 2012-08-10

**Authors:** Na Gao, Mingyi Wu, Shao Liu, Wu Lian, Zi Li, Jinhua Zhao

**Affiliations:** 1 State Key Laboratory of Phytochemistry and Plant Resources in West China, Kunming Institute of Botany, Chinese Academy of Sciences, Kunming 650201, China; Email: gn2008.happy@163.com (N.G.); mingyiwu_tju@yahoo.com (M.W.); lw3662410@yahoo.com.cn (W.L.); Lizi_08@126.com (Z.L.); 2 School of Pharmaceutical Sciences, Central South University, Changsha 410078, China; Email: liushao999@hotmail.com; 3 Xiangya Hospital, Central South University, Changsha 410008, China

**Keywords:** sea cucumber, fucosylated chondroitin sulfate, glycosaminoglycan, acylation, anticoagulant activity

## Abstract

Fucosylated chondroitin sulfate (FuCS), a kind of complex glycosaminoglycan from sea cucumber, has potent anticoagulant activity. In order to understand the relationship between structures and activity, the depolymerized FuCS (dFuCS) was chosen to prepare its derivates by selective substitution at OH groups. Its *O*-acylation was carried out in a homogeneous way using carboxylic acid anhydrides. The structures of *O*-acylated derivatives were characterized by NMR. The results indicated that the 4-*O*-sulfated fucose residues may be easier to be acylated than the other ones in the sulfated fucose branches. But the *O*-acylation was always accompanied by the β-elimination, and the degree of elimination was higher as that of acylation was higher. The results of clotting assay indicated that the effect of partial *O*-acylation of the dFuCS on their anticoagulant potency was not significant and the *O*-acylation of 2-OH groups of 4-*O*-sulfated fucose units did not affect the anticoagulant activity.

## 1. Introduction

The fucosylated chondroitin sulfate (FuCS) extracted from sea cucumber is a kind of complex glycosaminoglycan, due to its sulfated fucose (Fuc) branches linked to position 3 of the β-D-glucuronic acid (GlcUA) residues [1,2,3,4,5]. This glycosaminoglycan has high anticoagulant properties, which may open new avenues for the development of antithrombotic agents [3,6,7,8].The sulfated polysaccharide also displays several other pharmacological activities, such as anti-human immunodeficiency virus (anti-HIV) activity [9], attenuation of renal fibrosis through a P-selectin-mediated mechanism [10]. Thus, there is more and more increasing interest in the biological actions of the sulfated polysaccharide. 

One of most interest centers on the relationship between the structures and anticoagulant activities of FuCS. Due to its heavily sulfated fucose side chains at position 3 of the GlcUA, the sea cucumber chondroitin sulfate shows potent anticoagulant activity [1]. Removal of the sulfated fucose side chains by mild acid hydrolysis, as well as desulfation, reduces its anticoagulant and antithrombotic activities to the same levels as mammalian chondroitin sulfate [1]. In a preliminary study, in order to minimize an undesirable effect of platelet aggregation [11], a low molecular weight derivative (12,500 Da) of the sea cucumber chondroitin sulfate was obtained after H_2_O_2_ depolymerization from* Stichopus japonicus* [5]. However, there was no data concerning the effect of OH groups on its activities currently available until now. 

In the literature, in order to investigate effects of OH groups on biological activities of the sulfated polysaccharides, several researchers have prepared the *O*-acylated derivatives by using the ammonium salts of the polysaccharides and various carboxylic acid anhydrides. For example, Petitou *et al.* [12] selectively introduced acyl groups into heparin and its fragments. The measurements of anticoagulant and anti-HIV activities of these compounds indicated that their anti-HIV activities did not differ markedly from that of heparin, although their anticoagulant activities were much lower [13]. *O*-Acylated low-molecular-weight carrageenans were prepared. The results indicated the decreasing effect of the substituted acyl group on anticoagulant activity [14]. In order to increase the density of the negative charge of κ-carrageenan, introduction of free carboxyl into low molecular weight κ-carrageenan was executed by reaction of monocarboxyl of succinic anhydride with hydroxyl groups [15]. However, there could be more challenges for the study on *O*-acylation of the FuCS, since the structure of the FuCS may be more complex than those of the other glycosaminoglycans.

Recently, we isolated a new fucosylated chondroitin sulfate from the body wall of the sea cucumber *Thelenota ananas* [4], with a 2-OH of β-D-GlcUA residues in the chondroitin sulfate-like backbones and average ~1.5 OH groups in the sulfated fucose branches. The FuCS and its depolymerized fragments have been found to have significant anticoagulant activities [2,3]. Moreover, the anticoagulant activity as measured by the activated partial thromboplastin time assay varies in proportion to the molecular weight following a logarithmic-like function [3].

In the present study, in order to evaluate the feasibility of *O*-acylation of the FuCS and further investigate effects of the OH groups on anticoagulant activities, *O*-acylated derivatives of depolymerized FuCS fragments were prepared according to classical *O*-acylation of glycosaminoglycan. In addition, effects of reaction conditions on the process were investigated and the structures of the synthesized derivatives were characterized by the NMR technology. Furthermore, anticoagulant activities of the synthesized derivatives were examined, and also compared with those of depolymerized FuCSs with the similar molecular mass.

## 2. Results and Discussion

### 2.1. Analysis of the *O*-Acylation Reaction

#### 2.1.1. Effects of Reaction Conditions on Degrees of Substitution

**Figure 1:**
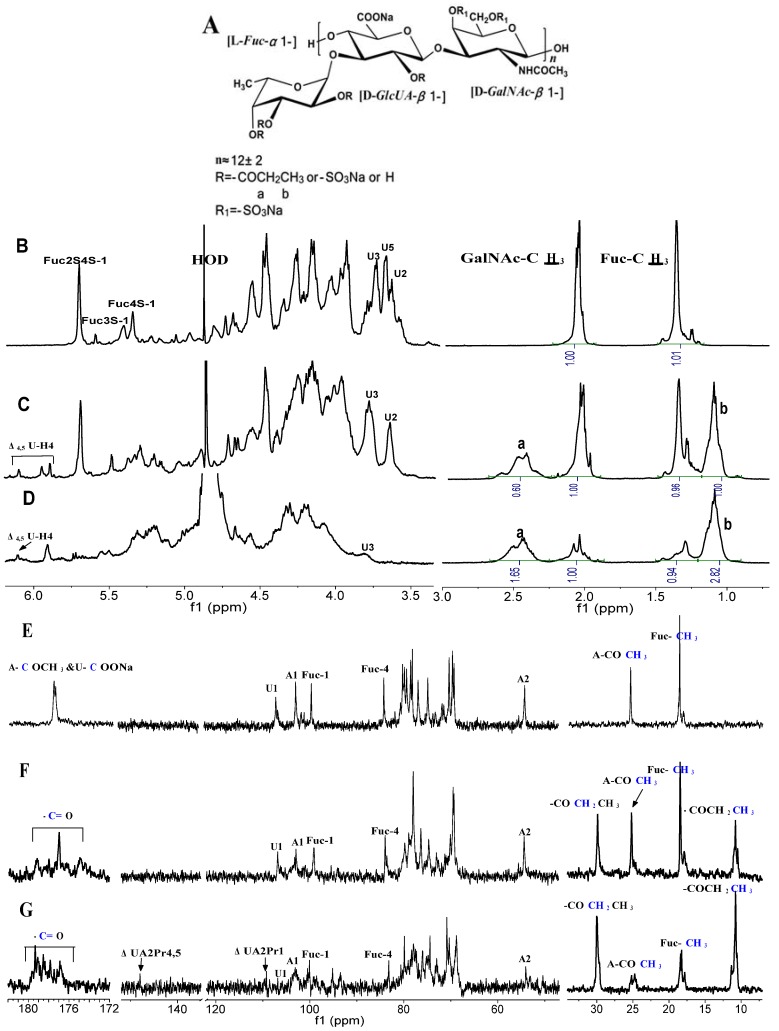
^1^H and ^13^C NMR spectra of the original dFuCS (**B, E**) and its different acylated degrees of *O*-propionyl derivatives (**C, F,** 40%; **D, G,** 95%). Chemical shifts are relative to external trimethylsilyl propionic acid at 0 ppm. The Panel A shows the structural units of the fucosylated chondroitin sulfate and its derivatives. Signals designate by “Fuc2S4S”, “Fuc3S”, “Fuc4S”, “A” and “GlcUA, or U” refer to those produced by 2,4-disulfated-fucose,3-sulfated-fucose, 4-sulfated-fucose residues, *N*-acetyl-β-D-galactosamin and β-D-glucuronic acid respectively. Then ΔU is α,β-unsaturated glucuronic acid residues. The legend *a* and *b* stands for the peaks of methyene and methyl group in the propionyl group respectively.

The structure of the depolymerized FuCS is similar to that of the native FuCS as previously described [2,3] (Figure 1), which has one OH group per disaccharide unit in the chondroitin sulfate backbones and average ~1.5 OH groups of α-L-fucose residue in the sulfated fucose branches. Thus, in theory, there are about 2.5 acyl groups per one mole of the corresponding monomer which could be introduced into the FuCS.

Generally, the degree of substitution for polysaccharides is expressed as moles of substituents per one mole of the corresponding monomer [16]. The degree of substitution can be precisely determined by ^1^H NMR spectroscopy (Figure 1).

In the work, the acylation of the dFuCS (Mw ~12,700 Da) proceeded with several anhydrides, such as acetic anhydride (C2), propionic anhydride (C3) and succinic anhydride (C4), and the reactions were carried out under different experimental parameters in the Table 1. Degree of acylation, and molecular weight of the products were showed in the Table 1.

**Table 1:** Degree of acylation, molecular weight of the *O*-acylated derivatives of dFuCS obtained with different carboxylic acid anhydrides under different experimental parameters and their anticoagulant activities.

Sample	Acylation ^a^	DMAP (mg)	Acid Anhydride	Temperature (°C)	Time (h)	Degree of substitution ^b^	Percentage of substitution (%)	*Mw* (Da) ^c^	APTT ^d^	
									μg/mL	U/mg
dFuCS-P35	Propionoylation	9.9	0.2 mL	40	24	0.88	35	6970	5.11	43
dFuCS-P40	Propionoylation	9.4	0.2 mL	40	48	1.00	40	6480	5.04	43
dFuCS-P84	Propionoylation	19.3	0.4 mL	40	48	2.08	84	4070	12.8	17
dFuCS-P95	Propionoylation	9.6	0.4 mL	60	48	2.38	95	3000	>44	<5
dFuCS-A37	Acetylation	10.2	0.3 mL	40	24	0.92	37	5830	6.11	36
dFuCS-S44	Succininoylation	9.8	300 mg	40	24	1.09	44	7700	4.47	49

^a ^The reactions were carried out at ~105 mg dFuCS, Mw ~12,700 Da; ^b ^The degree of substitution for polysaccharides is expressed as moles of substituents per one mole of the corresponding monomer. For example, the degree of substitution of the synthesized *O*-propionoyl derivative was determined by ^1^H NMR spectroscopy by comparing the amount of methylene groups of propanoyl linked to dFuCS with respect to the total amount of methyl groups in the *N*-acetylgalactosamine residue of dFuCS proton, and the degree of the *O*-acetyl dFuCS were obtained by comparing the amount of methyl groups on the acetyl groups to the amount of methy groups in the fucose branch,and similar methods were used for the *O*-succinoyl derivates; ^c ^The molecular weight (Mw) was examined by high-performance gel permeation chromatography (HPGPC); ^d ^The activity of dFuCS and *O*-acylated derivatives to prolong APTT was expressed by the concentration of each agent that is required to double the APTT (doubling APTT, μg/mL), and also were expressed as USP units/mg (heparin U/mg) using a parallel standard curve based on the USP heparin standard (212 units/mg) from Sigma (USA).

The propionoylation of the tetrabutylammonium salt of dFuCS proceeded successfully (Table 1) using propionic anhydride as acylating agent in the presence of a catalytic amount of DMAP at 60 °C. The highest degree of substitution of the synthesized *O*-propionoyl derivative was about 2.38, which was determined by ^1^H NMR spectroscopy by comparing the amount of methylene groups of propionoyl linked to dFuCS with respect to the total amount of methyl groups in the *N*-acetyl-β-D-galactosamine (GalNAc) residue of dFuCS (Figure 1).The result indicated that most of total OH groups were introduced into propionoyl groups, since the percentage of degree of substitution was ~95% after 48 h at 60 °C (Table 1). Under similar reaction conditions, Petitou *et al.* [12] successfully introduced acyl groups into other glycosaminoglycans, such as heparin, dermatan sulfate, and chondroitin sulfate.

The use of various amounts of acid anhydride or DMAP, different reaction temperature and time gave derivatives substituted to different degrees of substitution from 35% to 95% (Table 1). Comparison with the reaction conditions of the *O*-propionoyl derivative with the higher degree of substitution (~95% *vs.* 84%, 84% *vs.* 40%), the higher degree of substitution could be attributed to the reaction temperature (60 °C *vs.* 40 °C) and the amount of acid anhydride (0.4 mL *vs.* 0.2 mL), respectively (Table 1). But the yields of the products with higher degree of substitution were lower (~62% for 35% degree of substitution, *vs.* ~30% for 95% degree of substitution), which could be attributed the hydrolysis and breaking of the polysaccharide to smaller species.

**Figure 2:**
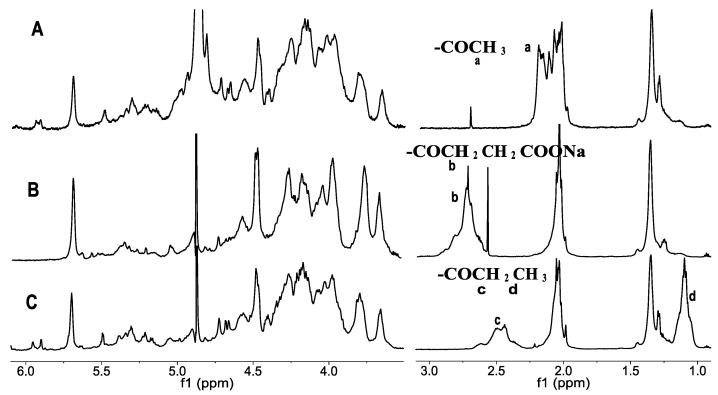
Part of ^1^H NMR of the *O*-acetyl fucosylated chondroitin sulfate (**A**), *O*-succinic acyl dFuCS (**B**) and *O*-propionyl dFuCS (**C**).

According to the established conditions, the products with different carboxylic acid anhydrides such as acetic anhydride, succinic anhydride, and propionic anhydride were prepared (Table 1, Figure 2). The degrees of acylation were about 35%–45% (Table 1, Figure 2), that is to say, about one acyl group could be introduced to the corresponding trisaccharide repeating monomer. The results indicated that these different carboxylic acid anhydrides did not affect the degrees of substitution of products under the similar conditions.

**Figure 3:**
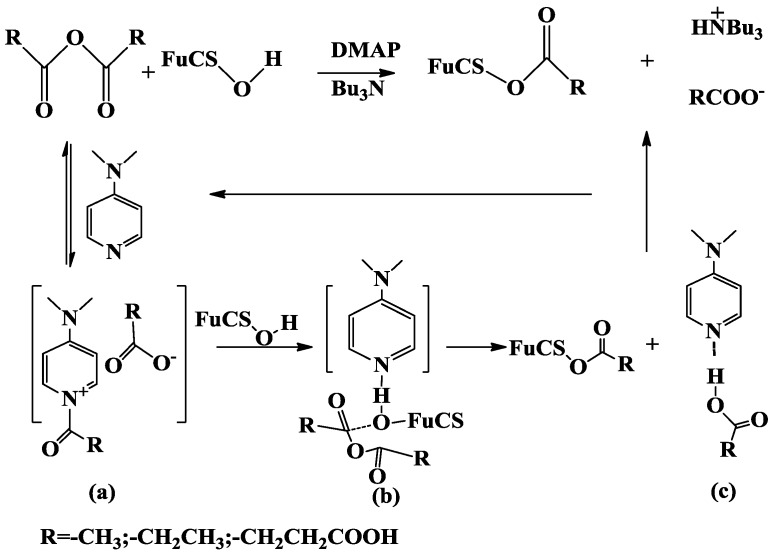
Proposed mechanism of the DMAP-catalyzed acylation of the fucosylated chondroitin sulfate.

In the study, the degree of acylation was only 30%–45% under the conditions of a catalytic amount of DMAP (9.4 mg) and 40 °C for 24 h (Table 1), *i.e.*, and only one acyl group was introduced into the corresponding monomer. But when the reaction temperature was increased to 60 °C, the hydroxyl groups (~2.5 per sugar unit) were almost acylated (95%). This difference could be related to the three dimensional structures of the native dFuCS and the steric hindrance between the bulky sulfate groups and the hydroxyl groups linked to the fucose branches. Thus, about one hydroxyl group was acylated and given the maximum degree of acylation of 30%–45% as indicated in the experimental result (Table 1). Conformation analysis of using a computer based on X-ray data, indicated the possibility that, in the molecular model of sulfated polysaccharide, the foregoing sulfate group and the neighboring hydroxyl group approached each other to form a hydrogen bond [17], which could result in difficulty of the acylation. Furthermore, the *O*-acylation of dFuCS by carboxylic acid anhydrides in the presence of DMAP as a catalyst could obey a possible nucleophilic catalysis mechanism reported in the literature (Figure 3) [18,19]. DMAP is a catalyst of outstanding utility in a variety of group-transfer reaction, such as the acylation of alcohols [18]. The reaction proceeds initiated via acylpyridiniumintermediate (*a* in Figure 3). In the nucleophilic addition of polysaccharide (FuCS) toward the formation of a ternary complex (*b* in Figure 3) containing DMAP, acetic anhydride and polysaccharide, which is common to both the nucleophilic and the general base catalysis pathway, then the carboxylate anion (RCOO^−^) abstracts the proton of polysaccharides. The reaction with polysaccharides produces acetates (RCOOFuCS) and the carboxylate salt (*c* in Figure 3) of DMAP via transition state *b*. This step is thought to be the rate-determining step. In the presence of a stoichiometric amount of auxiliary base such as tributylamine, DMAP is effectively regenerated. So if the amounts of the DMAP or the temperature are improved, it is easier for the agents to cross the energy barrier and will promote the rate of acylation.

#### 2.1.2. Effects Reaction Conditions on the Glycosidic Bond Cleavage

However, during the process, decreasing of the molecular weight (Mw) of the acylated products was observed (Table 1). Table 1 showed that the Mw of synthesized derivatives were significantly lower than that of their original dFuCS (~12,700 Da). Thus, the degradation of the dFuCS took place during the acylation reaction. Moreover, comparison with the molecular weights of the acylated products by the different reaction conditions, for example, the addition of amount of DMAP and tributylamine or increasing the temperature, indicated that the decreasing of the Mw could bedependent of the various parameters. 

**Figure 4:**
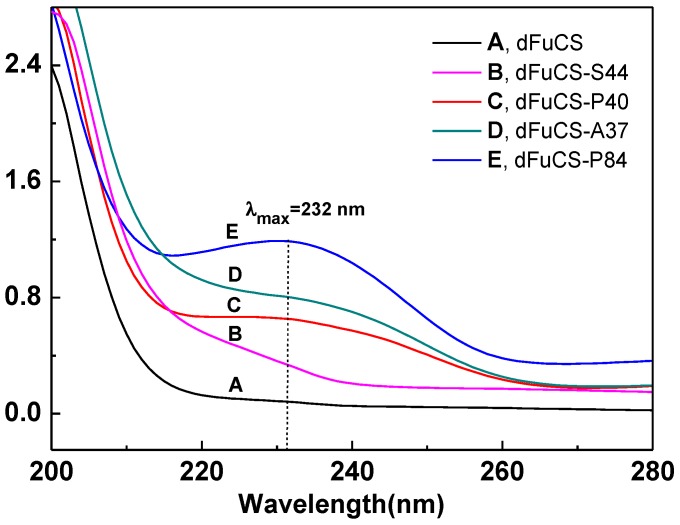
UV spectra of dFuCS (**A**),* O*-succinoyl (**B**), *O*-propionoyl (**C**, 40%; **E**, 84%), and *O*-acetyl (**D**) derivatives.

The ultraviolet spectra of the products (Figure 4), compared with that of the original dFuCS, showed that their maximum wavelengths (λ_max_) appeared at ~232 nm and the absorptions of products with lower molecular weight were stronger, corresponding to generating of unsaturated double bonds. Moreover, in the Figure 1C,D, the signal at about 6.0–6.1 ppm can be readily assigned to the proton H-4 of α,β-unsaturated carboxylic acids (∆4,5-glucuronic acid) in the ^1^H NMR spectrum. Thus, this polysaccharide may be depolymerized to the formation of α,β-unsaturated carboxylic acids by a β-elimination mechanism [20]. This degradation could be related to the catalytic agents just as DMAP and tributylamine. In the process, carboxyl groups of GlcUA units can be amidated by the DMAP and tributylamine, which becomes easy to be attacked, then GalNAc (β1→4) GlcUA linkages of the dFuCS can break by the β-elimination mechanism in the presence of the DMAP and tributylamine as two suitable proton acceptors.

### 2.2. Structural Analysis of Products by NMR

**Figure 5:**
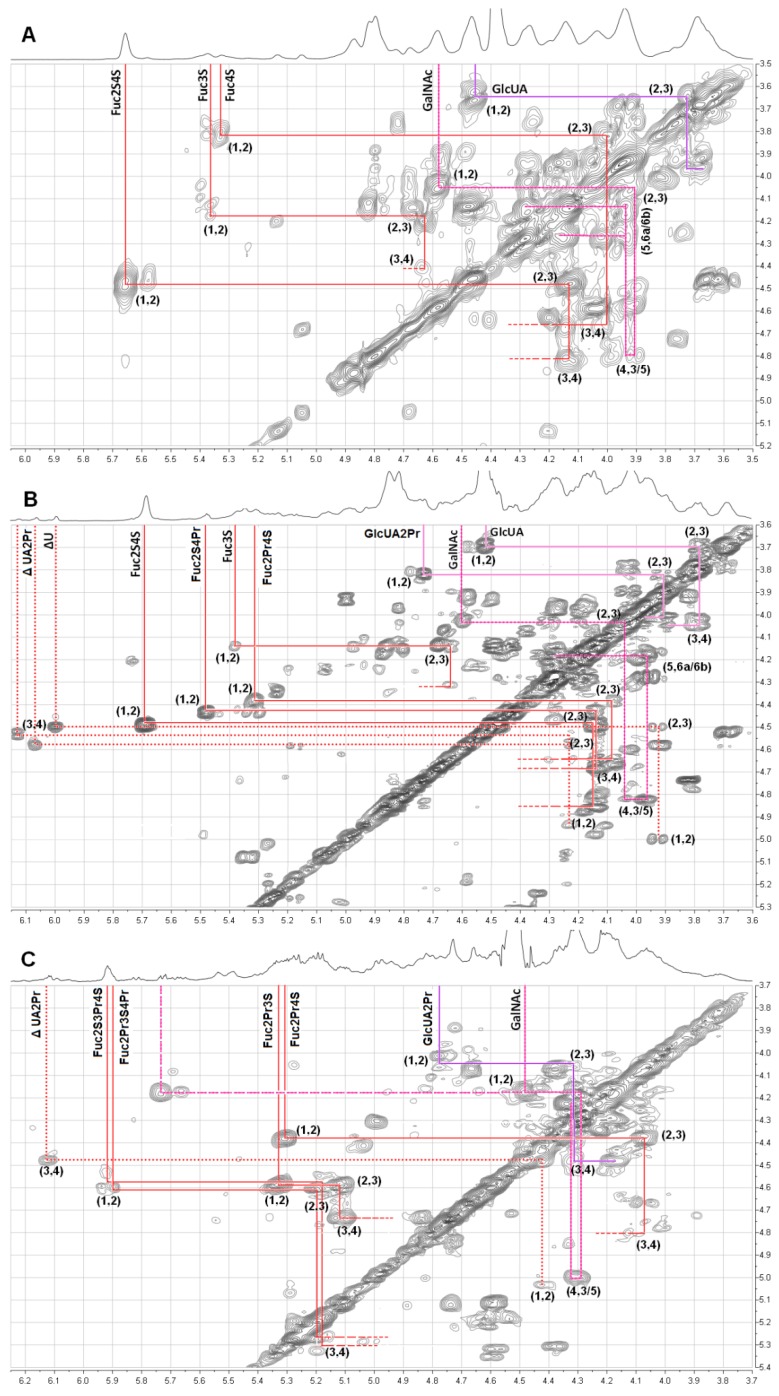
^1^H-^1^H COSY spectra of the dFuCS (**A**), its *O*-propionoyl derivativesdFuCS-P40 (degree of acylation, 40%) (**B**), and dFuCS-P95 (degree of acylation, 95%) (**C**), signals designate by “GalNAc4S6S” and “GlcUA” refer to those produced by 4,6-disulfated-acetyl-galactosamine, and β-D-glucuronic acid, respectively; whereas those of their corresponding *O*-acylated derivatives are labeled “Fuc2S3Pr4S”, “Fuc2S4Pr”, “Fuc2Pr3S4Pr”, “Fuc2Pr3S”, “Fuc2Pr4S” and “GlcUA2Pr”, respectively. Then ΔU, and ΔUA2Pr are the two kinds of α,β-unsaturated glucuronic acid residues.

The structures of the *O*-acylated derivatives were further studied by ^1^H and^ 13^C NMR spectroscopy. The 1D and 2D NMR spectra of the original dFuCS and its synthesized *O*-propionoyl derivative were shown in Figure 1 and Figure 5. The chemical shifts in Table 2 were based on the interpretations of the COSY spectra (Figure 5).

In the Figure 1C,D, two signals were present at 1.0–1.2 ppm and 2.3–2.6 ppm region, corresponding to the methyl and methylene protons of *O*-propionoyl FuCS. Interestingly, the H2 and H3 signals of β-D-glucuronic acid residue after the *O*-acylated process are at 3.82 ppm and 3.91 ppm (Figure 1, Table 2), respectively, shifted downfield by 0.2~0.3 ppm, according to trace the corresponding spin systems using the ^1^H-^1^H COSY (Figure 5). The result suggested that this may be attributed to effects of carbonyl of the propionoyl group at 2-position of β-D-glucuronic acid residues (GlcUA2Pr).

In addition, the ^1^H-^1^H COSY spectrum (Figure 5) showed that three signals with ~6.0–6.1 ppm in 1D NMR (Figure 1C) can be readily assigned to the proton H-4 of 4,5-unsaturated glucuronic acids (ΔUA). The proportion ∆4,5-glucuronic acid residues in the non-reducing end to fucose residues was about 7:100, according to integration of H4 resonances of the ΔUA and H1 resonances of the fucose residues in the one-dimensional spectrum (Figure 1C). The results suggested that the degradation may take place by β-elimination mechanism. 

In the ^1^H spectrum (Figure 1), the signals of synthesized *O*-propionoyl derivatives are not easy to find due to overlap of H signals among three sugar residues, but the carbonyl carbon signals is easily seen in the ^13^C spectrum. The carbonyl carbon signal of ester linkage was observed at the shift of ~178–179 ppm. There are signals at ~10.7 and ~29.9 ppm, respectively, corresponding to the methyl and methylene carbons of the propionoyl residue. Additionally, the signals at 140–150 ppm, ~110 ppm can be assigned to the unsaturated C of 4,5-unsaturated glucuronic acids, and C1 in their reducing ends, respectively. The data further confirmed that β-elimination had taken place.

Overall, comparison with the structures of their parent compound indicated that the structural characters of synthesized derivatives may not significantly change, such as the structure of the polyanionic backbone, but the degradation took place by the β-elimination mechanism.

Confirmation of acylated substitution at position is not easy due to the complex structure of the dFuCS with distinguishable patterns and proportions of sulfate substitution in the fucose branches [1,2,21]. However, priority of substitution of different OH in sugar unit could be elucidated. The chemical shifts of the anomeric protons of the 2,4-di-sulfated fucose (α-L-Fuc2S4S), 3-, and 4-mono-*O*-sulfated fucose (α-L-Fuc3S, α-L-Fuc4S) residues can be readily assigned according to comparison of the signals before and after the modification (Figure 5). 

In the ^1^H-^1^H COSY spectrum of the *O*-propionoyl dFuCS with ~40% degree of substitution (Figure 5B), the chemical shifts of the anomeric protons of the α-L-Fuc2S4S and α-L-Fuc3S residues are 5.68 and 5.38 ppm (Table 2, Figure 5A), respectively, which were in good agreement with those of original dFuCS in our previous study [2]. In addition, the others signals of the two kinds of fucose residues were not shifted, which clearly indicated that the OH groups of α-L-Fuc2S4S and α-L-Fuc3S residues were not introduced into propionoyl groups. In other words, the 3-OH groups of the α-L-Fuc2S4S residues and the 2-OH, 3-OH groups of the α-L-Fuc3S residues may be difficult to be acylated, possibly due to their special sulfation patterns. However, after the modification, the H-2 signal of the α-L-Fuc4S residues (δ_H-2_ = 4.34) was shifted downfield by ~0.5 ppm, which resulted from the propionoyl groups at the 2-position of α-L-Fuc4S residues (α-L-Fuc2Pr4S). Additionally, in the Figure 5B, the signals of the propionoyl groups at the 4-position of α-L-Fuc2S residues (α-L-Fuc2S4Pr) were observed, although the anomeric protons of α-L-Fuc2S and α-L-Fuc2S4S residues before the modification were overlap to not be discerned. Partial acylation of 2-OH of β-D-GlcUA residue in the chondroitin sulfate-like backbones took also place, but the degree of substitution were slight. These results suggested that the propionoyl groups may be preferentially introduced to the OH of the α-L-Fuc4S residues, slightly to those of α-L-Fuc2S in the fucose branches under above experimental conditions (Figure 5B).

**Table 2:** Proton chemical shifts of residues in the dFuCS and its *O*-propionoylated derivatives dFuCS-P40 (degree of acylation, 40%), dFuCS-P95 (degree of acylation, 95%) ^a^.

Compounds	Unit	Chemical Shift ( *ppm*)						
		H-1	H-2	H-3	H-4	H-5	H-6	Ac
dFuCS	β- D-GalNAc4S6S	4.58	4.05	3.93	4.82	3.97	4.16, 4.25	2.05
	β- D-GlcUA	4.46	3.64	3.73	3.93	3.67	/	/
	α- L-Fuc2S4S	5.66	4.47	4.13	4.82	4.87	1.35	/
	α- L-Fuc4S	5.33	3.82	4.00	4.80	4.86	1.35	/
	α- L-Fuc3S	5.36	4.14	4.63	4.40	4.51	1.25	/
dFuCS-P40	GalNAc4S6S	4.67	4.13	4.05	4.83	3.96	4.17, 4.28	2.03
	GlcUA	4.52	3.68	3.79	4.03	3.88	/	/
	GlcUA2Pr	4.73	3.82	3.91	4.02	3.95	/	/
	ΔUA	4.99	3.92	4.50	5.99	/	/	/
	ΔUA2Pr	4.94	4.23	4.52	6.12	/	/	/
	α- L-Fuc2S4S	5.68	4.49	4.14	4.85	4.84	1.36	/
	α- L-Fuc2S4Pr	5.47	4.44	4.13	4.79	4.87	1.36	/
	α- L-Fuc2Pr4S	5.31	4.34	4.07	4.63	4.86	1.35	/
	α- L-Fuc3S	5.38	4.14	4.64	4.30	4.33	1.28	/
dFuCS-P95	GalNAc4S6S	4.67	4.13	4.05	4.83	3.96	4.17, 4.28	2.03
	GlcUA	4.52	3.68	3.79	4.03	3.88	/	/
	GlcUA2Pr	4.73	3.82	3.91	4.02	3.95	/	/
	ΔUA2Pr	5.03	4.40	4.48	6.12	/	/	/
	α- L-Fuc2S3Pr4S	5.92	4.53	5.17	5.29	4.31–4.33	1.30–1.33	/
	α- L-Fuc2Pr3S4Pr	5.89	4.59	5.21	5.27			/
	α- L-Fuc2Pr3S	5.33	4.58	5.12	4.73			/
	α- L-Fuc2Pr4S	5.31	4.38	4.07	4.80			/

^a^ See the legend of the Figure 6 for additional information about the table.

However, the COSY spectrum of the *O*-propionoyl dFuCS with ~95% degree of substitution (Figure 5C) showed that the signals of Fuc and GlcUA residues became more complex. These H-2 and H-3 signals of GlcUA residues in the product were shifted downfield by ~0.2 ppm, which indicated that their 2-OH may be completely acylated. Besides remaining of OH in minor Fuc2Pr4S and Fuc2Pr3S, others hydroxyl groups were acylated, whose corresponding signals were significantly shifted downfield (Figure 5C).

### 2.3. Anticoagulant Activity

**Figure 6:**
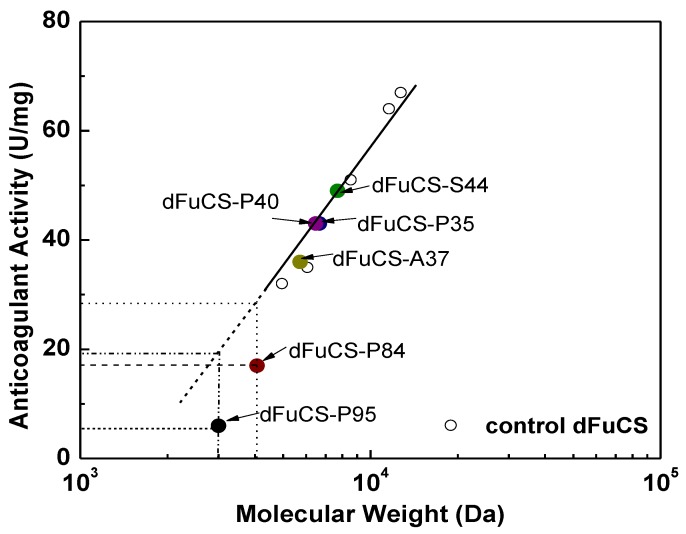
Effect of acylation on the anticoagulant activity.

Anticoagulant activity of *O*-acylated derivatives of the dFuCS from the sea cucumber *T. ananas* were assessed by measuring the activated partial thromboplastin time (APTT), and compared with the same activities of the dFuCSs, unfractionated heparin, and a low molecular weight heparin (LMWH, enoxaparin) from mammalian. The APTT assays (summarized in Table 1, Figure 6) indicated that five control dFuCS have significant anticoagulant activity (Figure 6), comparable with that of LMWH (32 U/mg), but lower than that of unfractionated heparin (212 U/mg). The Figure 6 showed that the anticoagulant activity varies of the five control dFuCS in proportion to the molecular weight following a logarithmic-like function according to an empirical formula [3]. The TT and PT assays were also carried out but none of the dFuCS and its *O*-acylated derivatives showed any effects on TT and PT at the concentrations (4–64 μg/mL) tested. The results were consistent with that from previous studies [2,3].

Interestingly, compared with the activity of control dFuCS with the similar molecular mass, partial *O*-acylation of the dFuCS with about 40% degrees of substitution did not affect their anticoagulant potency (Figure 6), although the dFuCS were substituted with different groups, such as acetic anhydride (C2), propionic anhydride (C3) and succinic anhydride (C4). Furthermore, comparison of the NMR data before and after the modification suggested that the *O*-acylation of 2-OH groups of 4-*O*-sulfated fucose units did not affect its anticoagulant activity. However, as the degree of *O*-acylation of the dFuCS increased, the activity decreased (Table 1, Figure 6). At 95% degree of *O*-acylation, the product had much low anticoagulant potency (<5 U/mg), about lower 2-fold than the dFuCS with similar molecular mass (Figure 6). But it is still difficult to completely rule out other causes such as molecular size, since the dFuCS with the low molecular weight (<4000 Da) may also have very low anticoagulant activity [3].

## 3. Experimental Section

### 3.1. Preparation of *O*-Acylated Derivatives of Depolymerized FuCS

A low molecular weight fragment of glycosaminoglycan from the sea cucumber *T. ananas* was prepared by free radical depolymerization [4]. The *O*-acylated derivatives of this depolymerized fragments (dFuCS) were prepared by treating their tetrabutylammonium salts with several anhydrides, such as acetic anhydride (C2), propionic anhydride (C3) and succinic anhydride (C4), according to modification of the method described previously [12].

In order to establish the protocol, several experiments were carried out as Table 1. The conditions of *O*-acylation are as follows, briefly, taking the *O*-propionoyl acylation as an example. The tetrabutylammonium salt (180 mg) was dissolved in anhydrous *N*,*N*-dimethylformamide (DMF), cooled to 0 °C, then 4-(*N*,*N*-dimethylamino) pyridine (DMAP) (9 mg), propionic anhydride (200 μL), and tributylamine (360 μL) were successively added in single portion. And the reaction was allowed to proceed under nitrogen at 40 °C for 48 h. After cooling to 0 °C, 5% NaHCO_3_ was gradually added to remove the superfluous anhydride, and the solution was stirred at 25 °C for another 48 h. Excess NaHCO_3_ was eliminated by the addition of 1 mol/L HCl until a pH 4.0 was reached, and then readjusted to pH 7.0 with 1 mol/L NaOH. 20% Cold absolute ethanol (v/v) was added with stirring. The sample was allowed to sit overnight at 4 °C to afford precipitate. The precipitate was recovered by centrifugation, which was dissolved in water, and passed through a column 35 × 2.5 (170 mL) of Dowex 50W × 8 (H^+^) cation exchange resin and 60 mL eluent was recovered. The acid was neutralized to pH 7.0 with 0.1 M NaOH. The raw products were purified by dialysis with MWCO 1000 Da against distilled water for 48 h. After lyophilization, the *O*-propionyl FuCS derivative (65 mg) was obtained as an off-white powder. In each case, selective *O*-acylation was performed in the presence of different carboxylic acid anhydrides with a catalytic amount of DMAP as the above process.

### 3.2. Analysis of Molecular Weight

In order to investigate the changing of molecular weight during the process of acylation reaction, the molecular weight (weight-average molecular mass, Mw) of the products during the course of the reaction were examined by high-performance gel permeation chromatography (HPGPC) using an Agilent technologies1200 series (Agilent Co., USA) apparatus equipped with a Shodex OH-pak SB-804 HQ column (8 mm × 300 mm) as described previously [4] .Specifically, the molecular weights of products were calculated using the standard curve by GPC software, which was dependent on the elution times of standard dextran oligosaccharides.

### 3.3. Spectrometry Analysis

The ultraviolet (UV) analyses were performed with an UV-2450 UV-Vis spectrophotometer (Shimadzu, Japan). All samples were dissolved in purified water at a ~0.8 g/L concentration.

NMR analyses were performed at 20 °C with a Bruker Avance 500 spectrometer of 500 MHz equipped with ^13^C/^1^H dual probe in the FT mode as described previously [22]. All lyophilized samples were dissolved in deuterium oxide (D_2_O, 99.9% D) at a 10–15 g/L concentration. The NMR experiments were recorded with a spectral width of 12,335.5 Hz, an acquisition time of 1.33 s, a pulse width of 7.8 s, a relaxation time of 2 s and a number of 16 scans. The HOD signal was presaturated by a presaturation sequence. All chemical shifts are relative to internal trimethylsilyl-propionic acid (TSP) sodium salt.

### 3.4. Anticoagulant Activity

The activated partial thromboplastin time (APTT), prothrombin time (PT), and thrombin time (TT) were determined with a coagulometer (TECO MC-4000, Germany). All the reagents were purchased from TECO GmbH (Germany). PT reagent, TT reagent, and normal human plasma were reconstituted in 4 mL (or 1 mL for plasma) of distilled water, according to the instructions of the manufacturer. The glycosaminoglycans were dissolved in 20 mM Tris-HCl (pH 7.4) at various concentrations. For the APTT assay, 5 μL samples were mixed with 45 μL of normal human plasma, and incubated for 2 min at 37 °C. Then 50 μL of APTT reagent was added to the mixture, which was incubated for another 3 min at 37 °C. CaCl_2_ (50 μL) was then added, and the clotting time was recorded. For the PT and TT assays, 5 μL samples (or 10 μL for TT) were mixed with 45 μL (or 90 μL for TT) of normal human plasma and incubated for 2 min at 37 °C; 100 μL of PT (or 50 μL of TT) reagent was then added and the clotting time was recorded.

## 4. Conclusion

In summary, the partially *O*-acylated derivatives of a depolymerized fucosylated chondroitin sulfate from *T. ananas* selectively substituted at OH groups were prepared, depending on experimental conditions. The structures of *O*-acylated derivatives were characterized by the NMR technology. The OH groups of the 4-mono-*O*-sulfated fucose residues may be more priority to be acylated than the other ones in sulfated fucose branches, possibly due to the steric effect and the hydrogen bond between the sulfate and the hydroxyl. But the degradation took place during the acylation reaction by possible β-elimination mechanism. 

Comparison between *O*-acylated dFuCS and dFuCS with the similar molecular mass by free radical degradation suggested that partial *O*-acylation of the dFuCS with about 40% degrees of substitution did not affect their anticoagulant potency, and the effect of *O*-acylation of 2-OH groups of 4-*O*-sulfated fucose units on the anticoagulant activity was also not significant. Thus, *O*-acylated oligosaccharides can be used to constitute a library of compounds useful for elucidation of the structure-activity relationship. 
